# Continuing education for the prevention of mild cognitive impairment and Alzheimer’s-type dementia: a systematic review protocol

**DOI:** 10.1186/s13643-017-0553-0

**Published:** 2017-08-08

**Authors:** Nina Matyas, Stefanie Auer, Christoph Gisinger, Monika Kil, Filiz Keser Aschenberger, Irma Klerings, Gerald Gartlehner

**Affiliations:** 1 Department of Evidence-based Medicine and Clinical Epidemiology, Danube University Krems, Dr. Karl Dorrek Str. 30, 3500 Krems, Austria; 2 Department of Clinical Neurosciences and Preventive Medicine, Danube University Krems, Dr. Karl Dorrek Str. 30, 3500 Krems, Austria; 3 Center for Geriatric Medicine and Geriatric Nursing, Danube University Krems, Dr. Karl Dorrek Str. 30, 3500 Krems, Austria; 4 Department of Continuing Education Research and Educational Management, Danube University Krems, Dr. Karl Dorrek Str. 30, 3500 Krems, Austria; 5 RTI International, 3400 East Cornwallis Rd, Durham, NC 27740 USA

**Keywords:** Systematic review, Dementia, Alzheimer, Mild cognitive impairment, Prevention, Continuing education

## Abstract

**Background:**

Because of the enormous social and economic burden of disease, the prevention of mild cognitive impairment and Alzheimer’s-type dementia has become a major global public health priority. Studies show that cognitively stimulating activities during middle adulthood might have a protective effect on the brain by boosting the cognitive reserve. The aim of this review is to identify evidence investigating the effects of continuing education for the prevention of mild cognitive impairment and Alzheimer’s-type dementia in late life.

**Methods:**

Our approach employs a two-stage design: First, we will conduct a systematic review to assess the preventive effects of continuing education on mild cognitive impairment and Alzheimer’s-type dementia. Second, because we expect to find few studies, we will perform a review of systematic reviews on leisure activities that mimic formal continuing education to determine their effects on the prevention of mild cognitive impairment and Alzheimer’s-type dementia. We will search electronic databases (e.g., MEDLINE, PsycINFO, EMBASE, CENTRAL, CINAHL, and Scopus) for published studies and gray literature databases (e.g., trial registries) for unpublished studies.

Two authors will independently screen abstracts and full-texts using pre-defined eligibility criteria, select studies, extract data, and assess the quality of included studies or reviews. Outcomes of interest include the incidence of mild cognitive impairment or Alzheimer’s-type dementia, quality of life, functional capacity, and psychological wellbeing. Intermediate outcomes are cognitive (test) performance, cognitive functioning, and social inclusion. The review team is a multidisciplinary group consisting of methodological experts and dementia, geriatrics, and continuing education researchers.

**Discussion:**

We anticipate that our review will highlight serious gaps in the current evidence. Results will build the basis for further research regarding the relation of continuing education and cognitive decline and dementia.

**Systematic review registration:**

PROSPERO CRD42017063944

**Electronic supplementary material:**

The online version of this article (doi:10.1186/s13643-017-0553-0) contains supplementary material, which is available to authorized users.

## Background

### Rationale

As a consequence of the rapidly aging world population, neurocognitive disorders such as mild cognitive impairment or Alzheimer’s-type dementia have become a major public health challenge. The World Health Organization (WHO) has designated the prevention and control of neurocognitive disorders as a public health priority [1].

Alzheimer’s-type dementia is the most common form of dementing illnesses. In 2015, 47.5 million people globally lived with Alzheimer’s disease or another closely related dementing illness [2]. The prevalence is projected to rise up to 135.5 million patients in 2050 [2].

Because of the progressing loss of independent functioning of patients with Alzheimer’s-type dementia, the social and economic burden of the disease is enormous. In 2016, the US economic burden associated with dementia was estimated to be 236 billion U.S. Dollars [3], the total global costs for dementia were 818 billion in 2015 [4].

The exact risk factors of neurocognitive disorders are largely unknown but the risk increases substantially with age. For example, the prevalence rate for Alzheimer’s disease surges from 3.5% in persons aged 75 or older to 46.3% in those aged 95 years or older [5].

Clinically, the onset of Alzheimer’s-type dementia is a slow process of cognitive deterioration. When the decline of cognitive functioning reaches a level that can be measured objectively, it is often referred to as mild cognitive impairment or mild neurocognitive disorder [6]. Petersen describes this as a “transitional period” between normal aging and the diagnosis of Alzheimer’s-type dementia. In this study, mild cognitive impairment refers to “amnestic” mild cognitive impairment (aMCI), which is defined as a condition where memory loss is predominant [7]. In approximately 32% of patients with aMCI, cognitive decline progresses to a degree that the ability of a person to perform everyday activities is significantly impaired. Such a state is called major neurocognitive disorder due to Alzheimer’s-type dementia [6, 8]. Based on the criteria of the Diagnostic and Statistical Manual (DSM-5), Alzheimer’s disease is characterized by a significant decline of intellectual abilities in one or more cognitive domains (learning and memory, language, executive function, complex attention, perceptual motor function, social cognition) outside the context of delirium [8].

The underlying risks for Alzheimer’s-type dementia are not yet thoroughly understood, and no curative treatment has been found [9]. Systematic reviews assessing risk factors indicate that low educational level, decreased physical activity, unhealthy diet, smoking, and alcohol abuse might be predictors of dementia [10–13]. Likewise, chronic medical conditions such as cardiovascular diseases, diabetes, obesity, cancers, depression, thyroid disorder, or genetic factors increase the risk of dementia [10]. Some studies, however, found a protective association of cognitively stimulating activities, such as learning a new language in middle age, with a slower cognitive decline during late life [14–18]. Such results underpin a theory called the “cognitive reserve hypothesis” [19, 20]. According to this theory, through every activity that stimulates the brain, the cognitive reserve gets boosted and the resistance towards any dementia-related brain pathology gets stronger [21]. A larger cognitive reserve acquired by continuing education activities, thus, might protect against cognitive decline [21, 22].

For this systematic review, we define continuing education as structured learning activities and programs provided by formal and non-formal educational institutions for persons beyond the age of compulsory schooling. These activities are designed to help individuals satisfy learning needs and interests, to enrich knowledge, to develop and improve abilities and skills, and to foster personality, social competences, families, networks, health, and professional life. Continuing education is voluntary and based on topics and courses that are not directly connected to any special job position or vocational training [23–28].

To date, the preventive effect of continuing education on cognitive impairment and Alzheimer’s-type dementia has not been assessed in an objective and systematic way.

### Objectives

The purpose of our review is to summarize the evidence investigating the effects of continuing education on the development of cognitive impairment and Alzheimer’s-type dementia. Based on discussions with experts in the fields of neurocognitive disorder, aging, and continuing education, we designed the following five key questions that will guide our systematic review:

1. In adults 45 years of age or older with normal cognition or merely subjective cognitive impairment, does continuing education lead to a reduction in the risk of mild cognitive impairment or Alzheimer’s-type dementia compared with no continuing education?

1. (In case no evidence on continuing education is available): In adults 45 years of age or older with normal cognition or merely subjective cognitive impairment, do leisure activities lead to a reduction in the risk of mild cognitive impairment or Alzheimer’s-type dementia compared with no continuing education?

Key question 2: What are the potential harms of continuing education?
Key question 3: Do benefits and harms differ by subgroups based on age, sex/gender, race or ethnicities, level of education, or duration of intervention?
Key question 4: What is the optimal age to start continuing education to prevent mild cognitive impairment or dementia?

## Methods

This systematic review protocol has been designed in accordance with the Preferred Reporting Items for Systematic Reviews and Meta-Analyses-Protocol (PRISMA-P) statement [29] (see Additional file 1). Our protocol is registered on PROSPERO (international prospective register of systematic reviews) (registration number CRD42017063944).

### Study design

**Fig. 1:**
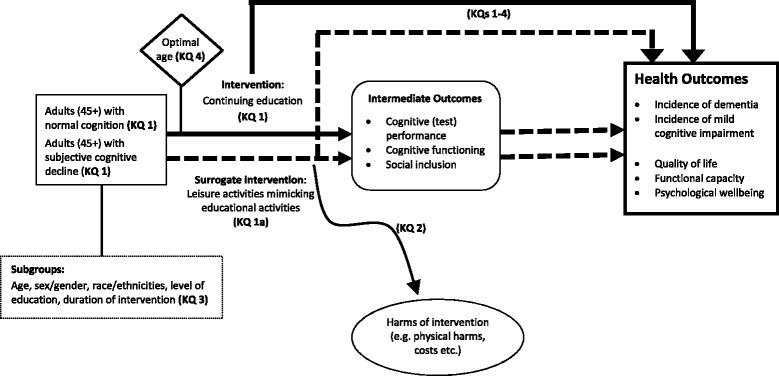
Analytic framework for continuing education to prevent mild cognitive impairment and Alzheimer’s-type dementia

Figure 1 presents an analytic framework of the effects of continuing education on relevant health outcomes. Because we expect few studies that assess formal continuing education, we will also include leisure activities that mimic continuing education regarding content, but are not organized educational activities (e.g., learning a new language privately versus learning a new language as an organized educational activity). Because leisure activities are not our primary focus of interest but can be considered as surrogate interventions in some circumstances, we will address our questions of interest with two different methodological approaches:

1. We will employ a systematic review of primary studies to assess the preventive effects and potential harms of continuing education provided by formal and non-formal institutions.
2. We will use a review of systematic reviews to determine the preventive effects and potential harms of related leisure activities.

A review of systematic reviews is a synthesis of evidence from multiple systematic reviews [30]. For both approaches, we will employ the same criteria for populations, control interventions, outcomes, timing, and settings as outlined below. Criteria for interventions and eligible study designs will be different.

### Eligibility criteria

We specified our inclusion and exclusion criteria based on the population, intervention, comparators, outcomes, timing, and settings (PICOTS) identified through a topic refinement exercise with experts in the field (Table 1). We will exclude study designs without control groups to ensure that our pool of included studies can inform the causal link between the intervention and outcomes.

**Table 1:** Criteria for inclusion and exclusion of studies in the review

Criteria		
Category	Inclusion	Exclusion
Population	-Adults (45 years or older) without a clinical diagnosis of cognitive impairment; this includes people with subjective cognitive impairment	-People younger than 45 years -People with a clinical diagnosis of impaired cognition (e.g., MMSE < 24) -Populations comprised exclusively of patients with primary diseases with an increased risk for dementia such as Parkinson disease, HIV infection, multiple sclerosis, stroke, post traumatic brain injuries, infectious diseases, psychiatric conditions (e.g., alcohol abuse, drug abuse, major depressive disorder)
Subgroups	-Age -Sex/gender -Race/ethnicity -Level of education -Duration of intervention	
Geography	No limit	No limit
Date of search	Searches will go back until 1990	
Settings	Community-dwelling adults	Institutionalized people, e.g., people in nursing homes
Interventions	For systematic review -All cognitive activities that are provided by formal and non-formal educational institutions -Classes/courses/trainings that are based on individual interests and that are attended voluntarily For review of systematic reviews -Leisure activities that are cognitively stimulating and mimic the content of continuing education but in an informal setting.	Formal (vocational) education and training, physical activities, topics and courses that are related to any special job position and/or occupation
Control interventions	No continuing education	Any educational activities and physical activities
Outcomes	Health outcomes - Incidence of dementia - Incidence of MCI - Psychological wellbeing - Functional capacity - Quality of life - Other relevant health outcomes Intermediate outcomes - Cognitive functioning - Cognitive (test) performance - Social inclusion	
Timing	Minimum duration of the intervention: 3 months Minimum follow-up time: 1 year	
Publication language	No language restrictions	
Study design	For systematic reviews -RCTs -Nonrandomized controlled trials -Prospective controlled cohort studies -Retrospective controlled cohort studies -Case-control studies -Nonrandomized studies must have a minimum sample size of 300 or more participants For review of systematic reviews -Systematic reviews and meta-analyses	-Case series -Case reports -Cross over trials -Nonsystematic reviews -Studies without a control group -Nonrandomized studies with fewer than 300 participants

Abbreviations: *MCI* mild cognitive impairment, *MMSE* Mini-Mental State Examination, *HIV* human immunodeficiency virus, *RCT* randomized controlled trial

#### Types of participants

We will include studies with adults (45 years or older) who do not have a clinical diagnosis of cognitive impairment or Alzheimer’s-type dementia. This population also includes adults with subjective cognitive impairment who do not fulfill the criteria for a clinical diagnosis of mild cognitive impairment.

Subgroups of interest are based on age, sex/gender, race or ethnicity, level of education, socio-economic status, and duration of intervention.

We will not include patients younger than 45 years. Furthermore, we will exclude populations with other severe health conditions that are likely to be associated with loss of cognitive function such as HIV (human immunodeficiency virus) infections, multiple sclerosis, stroke, post traumatic brain injuries, and psychiatric conditions (e.g., alcohol abuse, drug abuse, major depressive disorder).

#### Types of interventions

The systematic review will examine any intervention that can be categorized as “continuing education”. In accordance with our definition, we include all courses, classes, lectures, workshops, and trainings that are provided by institutions of formal and non-formal education. The eligible learning activities are based on topics that are not directly connected to any special occupation. Individuals engage in continuing education voluntarily and intentionally to satisfy their learning needs and interests and to develop or improve their abilities and skills [23–28]. Because the focus of this research is on the effects of cognitive activities, all continuing education involving a physical activity will be excluded.

For the review of systematic reviews, we include leisure activities that mimic the content of continuing adult education but in an informal setting. For example, we would include playing chess as a leisure activity if no studies on playing chess in a formal continuing education setting are available. We will not include leisure activities for which formal continuing education programs are unlikely (e.g., going to the cinema), or for which physical exercise is the main goal (e.g., jogging, yoga).

#### Types of comparators

Studies with any type of control group will be included.

#### Types of outcome measures

Our primary outcome of interest will be the incidence of mild cognitive impairment or Alzheimer’s-type dementia. Furthermore, we are interested in any health outcome such as psychological wellbeing, functional capacity, or quality of life. In the absence of evidence on health outcomes, we will include intermediate outcomes such as cognitive functioning, cognitive (test) performance, or social inclusion.

#### Timing

The minimum duration for any intervention is 3 months; the minimum follow-up time is 1 year.

#### Setting

We will include any communities and institutions that offer continuing education. We will exclude institutions such as retirement homes, hospitals, or nursing homes.

#### Types of studies

We will include studies with control groups only. Eligible for inclusion are randomized controlled trials, nonrandomized controlled trials, prospective controlled cohort studies, retrospective controlled cohort studies, and case-control studies.

The review of systematic reviews will include any systematic review or meta-analysis that addresses a question of interest. We define systematic reviews based on the Cochrane handbook as a literature review that attempts to collate all empirical evidence using (a) a clearly stated objective and pre-defined eligibility criteria, (b) an explicit reproducible methodology, (c) a systematic search, (d) an assessment of the validity of the findings of the included studies, and (e) a systematic presentation, and synthesis, of the characteristics and findings of the included studies [30].

### Information sources and literature search

We will systematically search, review, and analyze the scientific evidence for each key question. To identify articles relevant to each key question, we will begin with a Ovid MEDLINE search for eligible interventions using a combination of medical subject headings (MeSH®) and title and abstract keywords, limiting the search to human-only studies without applying any language limitations (see Additional file 2: Ovid MEDLINE search strategy). We will also search the Cochrane Library, Embase, PsycINFO, CINAHL (Cumulative Index to Nursing and Allied Health Literature), ALOIS (the Cochrane Dementia and Cognitive Improvement Group Specialized Register), and ERIC (Education Resources Information Center) using analogous search terms. We selected these databases based on preliminary searches and consultation with content experts. The search period will go back to January 1990. An experienced information specialist will perform all searches; the electronic Ovid Medline search strategy was peer-reviewed by another information specialist following the PRESS (peer review of the electronic search strategy) statement [31].

In addition, we will search for gray literature (i.e., unpublished studies) relevant to this review. Potential sources of gray literature include ClinicalTrials.gov, the World Health Organization’s International Clinical Trials Registry Platform, websites of relevant organizations, and dissertation databases (e.g., DART-Europe).

In an attempt to avoid retrieval bias, we will manually search the reference lists of landmark studies and background articles on this topic to look for any relevant citations that our electronic searches might have missed.

We will include studies that meet all the inclusion criteria and contain enough methodological information to enable us to assess risk of bias.

### Data abstraction and data management

Two authors will independently review all titles and abstracts identified through searches for eligibility against our inclusion/exclusion criteria. Studies marked for possible inclusion by either reviewer will undergo a full-text review. For studies without adequate information to determine inclusion or exclusion, we will retrieve the full text and then make the determination. All results will be tracked in an EndNote® X8 bibliographic database (Thomson Reuters, New York, NY).

We will retrieve and review the full text of all titles included during the abstract review phase. Two authors will independently review each full-text article for inclusion or exclusion based on the eligibility criteria described above. If both reviewers agree that a study does not meet the eligibility criteria, the study will be excluded. If the reviewers disagree, conflicts will be resolved by discussion and consensus or by consulting a third member of the review team. We will record the reason that each excluded full-text publication did not satisfy the eligibility criteria so that we can later compile a comprehensive list of such studies.

For studies that meet our inclusion criteria, we will abstract important information into evidence tables. We will design data abstraction forms to gather pertinent information from each article, including characteristics of study populations, settings, interventions, comparators, study designs, modifiable risk factors [32], methods, and results. Trained reviewers will extract the relevant data from each included article into the evidence tables. A second member of the team will review all data abstractions for completeness and accuracy.

### Risk of bias assessment

To assess the risk of bias (internal validity) of studies, we will use the Cochrane Risk of Bias Tool for randomized controlled trials [33] and the Risk of Bias in Non-randomized Studies of Interventions (ROBINS-I) tool for nonrandomized studies [34]. For the appraisal of systematic reviews of RCTs, we will use the AMSTAR (Assessing the Methodological Quality of Systematic Reviews) tool [35].

We will consider the risk of bias for each relevant outcome of a study.

Two independent reviewers will assess the risk of bias for each outcome in each study. Disagreements between the two reviewers will be resolved by discussion and consensus or by consulting a third member of the team.

### Data synthesis and statistical analysis

If we find three or more similar studies for a comparison of interest, we will consider quantitative analysis (i.e., meta-analysis) of the data from those studies. For all analyses, we will use random-effects models to estimate pooled effects.

To determine whether quantitative analyses are appropriate, we will assess the clinical and methodological heterogeneity of the studies under consideration following established guidance [36]. We will do this by qualitatively assessing the PICOTS of the included studies, looking for similarities and differences. For meta-analyses, we will assess statistical heterogeneity in effects between studies by calculating the chi-square statistic and the *I* ^2^ statistic (the proportion of variation in study estimates attributable to heterogeneity) [37, 38]. The importance of the observed value of *I* ^2^ depends on the magnitude and direction of effects and on the strength of evidence for heterogeneity (e.g., *p* value from the chi-squared test or a confidence interval for *I* ^2^). If we include any meta-analyses with considerable statistical heterogeneity, we will provide an explanation for doing so, considering the magnitude and direction of effects. We will also examine potential sources of heterogeneity using sensitivity analyses or analysis of subgroups. We plan to stratify analyses and/or perform subgroup analyses when possible and appropriate to examine clinical heterogeneity.

For any quantitative analyses, we will conduct sensitivity analyses including high risk of bias studies. Planned stratifications or categories for subgroup analyses include the subgroups listed in the analytic framework. When quantitative analyses are not appropriate (e.g., because of heterogeneity, insufficient numbers of similar studies, or insufficiency or variation in outcome reporting), we will synthesize the data qualitatively.

To assess publication bias, we will use funnel plots and Kendall’s tests.

We will assess the quality of evidence for individual comparisons and outcomes using the Grading of Recommendations, Assessment, Development and Evaluation (GRADE) approach [39].

## Discussion

To the best of our knowledge, this will be the first review to assess the potential benefits and harms of continuing education for the prevention of mild cognitive impairment and Alzheimer’s-type dementia. We anticipate that this review will identify serious gaps in the current evidence. Our results will build the basis for further research and highlight implications for practice. Through our multidisciplinary team, results can easily reach a variety of stakeholders and findings can be disseminated through many channels.

## Additional files

Additional file 1: PRISMA-P 2015 checklist. (DOCX 35 kb)

Additional file 2: Ovid MEDLINE search strategy. (DOCX 16 kb)

## Abbreviations

AD: Alzheimer’s disease
aMCI: Amnestic Mild Cognitive Impairment
AMSTAR: Assessing the Methodological Quality of Systematic Reviews
CEDEFOP: European Centre for the Development of Vocational Training
CENTRAL: Cochrane Central Register of Controlled Trials
DSM: Diagnostic and Statistical Manual of Mental Disorders
GRADE: Grading of Recommendations Assessment, Development and Evaluation
ICD: International Classification of Diseases
MCI: Mild Cognitive Impairment
MMSE: Mini-Mental State Examination
OECD: Organisation for Economic Co-operation and Development
PRISMA-P: Preferred Reporting Items for Systematic Reviews and Meta-Analyses extension for Protocols
UNESCO: United Nations Educational, Scientific and Cultural Organization
WHO: World Health Organization
