# Supplementary material for: Continuing education for the prevention of mild cognitive impairment and Alzheimer’s-type dementia: a systematic review protocol
Source: Syst Rev. 2017 Aug 8;6:157. doi: 10.1186/s13643-017-0553-0 (PMC5549351; doi:10.1186/s13643-017-0553-0)
Supplement: Supplementary file 2 — Ovid MEDLINE search strategy. (DOCX 16 kb) [file 13643_2017_553_MOESM2_ESM.docx]

**Draft MEDLINE search strategy**

Ovid MEDLINE(R) 1946 to April Week 2 2017, Ovid MEDLINE(R) Epub Ahead of Print April 24, 2017, Ovid MEDLINE(R) In-Process & Other Non-Indexed Citations April 24, 2017, Ovid MEDLINE(R) Daily Update April 24, 2017, Ovid MEDLINE(R) Versions

| **#** | **Suchen** | **Ergebnisse** |
| --- | --- | --- |
| 1 | Dementia/ or exp Alzheimer Disease/ | 115094 |
| 2 | Cognitive Dysfunction/ | 5277 |
| 3 | Cognition/ | 79988 |
| 4 | (dementia or alzheimer*).ti,ab. | 170540 |
| 5 | ((cognit* or memory or mental) adj3 (impair* or decline* or deficit* or reduc* or function*)).ti,ab. | 162068 |
| 6 | or/1-5 | 361785 |
| 7 | Primary Prevention/ | 16651 |
| 8 | prevent*.ti,ab. | 1184849 |
| 9 | (protect* or reduc* or delay* or improv*).ti. | 696919 |
| 10 | or/7-9 | 1823889 |
| 11 | 6 and 10 | 37914 |
| 12 | exp Dementia/pc or Cognitive Dysfunction/pc [Prevention & Control] | 4891 |
| 13 | 11 or 12 | 40708 |
| 14 | exp Education, Continuing/ | 60663 |
| 15 | ((continuing or adult) adj2 (education or training)).af. | 80026 |
| 16 | (lifelong learning or life-long learning).af. | 2342 |
| 17 | education/ or exp curriculum/ or exp education, distance/ or exp education, nonprofessional/ or exp educational measurement/ or exp international educational exchange/ or mentoring/ or exp schools/ or exp teaching/ | 495093 |
| 18 | Learning/ | 55530 |
| 19 | Students/ | 46492 |
| 20 | exp Educational Status/ | 47011 |
| 21 | (learn* or class or classes or course? or educat* or school* or train* or seminar* or tutor*).ti. | 561407 |
| 22 | education.fs. not exp Health Personnel/ed | 198387 |
| 23 | (cognitiv* adj2 (stimulat* or activit*)).ti. | 718 |
| 24 | or/14-23 | 1062887 |
| 25 | 13 and 24 | 3281 |
| 26 | exp animals/ not exp humans/ | 4388804 |
| 27 | 25 not 26 | 2507 |
| 28 | exp age groups/ not (exp aged/ or middle aged/) | 3666482 |
| 29 | 27 not 28 | 1864 |
| 30 | limit 29 to yr="1990 -Current" | 1831 |
| 31 | remove duplicates from 30 | 1732 |
